# Conditional deletion of *HDAC4* from collagen type 2α1-expressing cells increases angiogenesis in vivo

**DOI:** 10.1186/s10020-020-00154-6

**Published:** 2020-05-01

**Authors:** Lilan Gao, Shengchun Li, Xiaochun Wei, Guoqing Du, Dennis Wei, Lei Wei

**Affiliations:** 1 Tianjin Key Laboratory for Advanced Mechatronic System Design and Intelligent Control, School of Mechanical Engineering, Tianjin University of Technology, Tianjin, 300381 China; 2 National Demonstration Center for Experimental Mechanical and Electrical Engineering Education (Tianjin University of Technology), Tianjin, 300381 China; 3 Department of Orthopedics, Warren Alpert Medical School of Brown University / RIH, Coro West/402H, 1 Hoppin St, Providence, RI 02903 USA; 4 The Second Hospital of Shanxi Medical University, Taiyuan, 030001 China; 5 Shi’s Center of Orthopedics and Traumatology, Shuguang Hospital Affiliated to Shanghai University of TCM, Shanghai, 201203 China; 6 Northeastern University, Boston, MA 02115 USA

**Keywords:** Histone deacetylase 4, Growth plate, Mice, Chondrocytes, Angiogenesis

## Abstract

**Background:**

HDAC4 is a key regulator of chondrocyte hypertrophy and skeletal development, but it is not clear whether the increase in vascular invasion at growth plates is related to HDAC4 expression. To determine it, we investigated the relationship between HDAC4 and angiogenesis in both in vivo and in vitro models.

**Methods:**

HDAC4 was deleted in Col2α1-Cre; HDAC4^fl/fl^ mice. Growth of the *Col2α1-Cre;* HDAC4^d/d^ mice was compared with HDAC4^fl/fl^ mice at postnatal days 2, 4, 6, and 8. X-rays were taken to examine skeletal development. At postnatal days 14 and 21, mice were euthanized for specimen collection. Murine chondrocytes were isolated from the ventral parts of rib cages of 6-day-old mice (C57Bl/6) and transfected with a vector expressing HDAC4 as a fusion protein with green fluorescent protein (GFP). Relative expression levels of HDAC4, VEGF, and Hif1α were measured in these cells by Western blot, RT-qPCR, enzyme-linked immunosorbent, histology, and immunohistochemistry assays.

**Results:**

The *Col2α1-Cre*; HDAC4^d/d^ mice were markedly smaller compared with the control mice. At postnatal days 14 and 21, the *Col2α1-Cre*; HDAC4^d/d^ mice exhibited a shortened growth plate, a larger secondary ossification center, and stronger staining of CD31 and CD34 compared to control mice. The isolated chondrocyte cells exhibited a high transfection efficiency of HDAC4 which resulted in the detection of a significant decrease in VEGF and Hif1α levels compared with the control chondrocytes.

**Conclusions:**

HDAC4 expression in chondrocytes contributes to angiogenesis in the growth plate, and its absence in vivo negatively affects growth plates.

## Background

During endochondral ossification, mesenchymal cells differentiate into chondrocytes which eventually form a cartilage growth plate. Ossification begins when hypertrophic chondrocytes undergo programmed cell death and the resulting calcified cartilage is invaded by blood vessels, osteoblasts, osteoclasts, and mesenchymal precursors to form primary ossification centers. Within these centers, bone eventually replaces the disappearing cartilage. Degradation and remodeling of the cartilage matrix are essential for vascular invasion and angiogenesis, and this step is considered to be a crucial step in endochondral ossification. This process has been shown to be coordinately controlled by several signaling molecules, including HDAC4, fibroblast growth factor 2 (FGF-2), bone morphogenetic protein 2 (BMP2), vascular endothelial growth factor (VEGF), and Indian hedgehog (IHH) proteins (Chen et al. 2016b; Huang et al. 2016a, b).

Histone deacetylase 4 (HDAC4) is a key member of a family of tissue-specific class II HDAC proteins. HDAC4 is expressed in prehypertrophic chondrocytes in the growth plate and is a negative regulator of growth plate maturation by inhibiting matrix metalloproteinase (MMP) -13 expression and inhibiting the activity of runt-related transcription factor-2 (Runx2) which is essential for chondrocyte hypertrophy during endochondral bone formation (Vega et al. 2004; Li et al. 2014; Cao et al. 2014). We previously observed that expression of HDAC4 significantly decreased in hypertrophic chondrocytes present in the growth plates of newborn mice (Guan et al. 2011). While it is well established that HDAC4 is a key regulator of chondrocyte hypertrophy and skeletal development (Wang et al. 2014a, b), it is not clear whether the increase in vascular invasion at the hypertrophic zone and bone junction area is related to HDAC4 expression.

Other scholars investigated the relationship between HDAC4 regulation of HIF-1, VEGF and angiogenesis in cancer cell line (Geng et al. 2011; Ellis et al. 2009). Geng H et al. found that HDAC4 inhibition led to the reduction of HIF-1-mediated target gene expressions and activities in cancer cells under hypoxic condition. However HDAC4 inhibition had little effect on Hif1α under normal condition. And they suggested that not all HIF-1 target genes were down-regulated by HDAC4 inhibition (Geng et al. 2011). Ellis L et al. reviewed the influence of HIF-1α and VEGF on tumor angiogenesis and how HDACs played a critical role in HIF-1α transcriptional activity. The studies presented that HDAC4 played an important role in hypoxic induced angiogenesis, and that pharmalogical inhibition and shRNA against HDAC4 offered a new strategy in anti-cancer therapy through decreasing HIF-1α expression and inhibiting angiogenesis in tumor (Ellis et al. 2009). However the relationship of HDAC4 regulation of HIF-1, VEGF and vascular invasion at the hypertrophic zone and bone junction area remains unclear.

To examine the significance of chondrocyte-derived HDAC4 for angiogenesis at the hypertrophic zone and bone junction area, we investigated the relationship between HDAC4 and angiogenesis in both in vivo and in vitro models. Since global deletion of HDAC4 is lethal in mice (Vega et al. 2004), we generated a conditional knockout mouse model of HDAC4 to perform in vivo studies. For an in vitro model, HDAC4 was overexpressed as a fusion protein with green fluorescent protein (GFP) in chondrocytes. In these models, the role of HDAC4 in relation to angiogenesis and angiogenesis-related proteins was examined, respectively.

## Methods

### Generation of Floxed *HDAC4* animals

Approval for the animal experiments conducted in this study was obtained from the Institutional Animal Care and Use Committee at Rhode Island Hospital. *Col2α1-Cre* mice were mated to HDAC4^fl/fl^ (from Dr. Olson, University of Texas Southwestern Medical Center) animals to obtain HDAC4^fl/−^, *Col2α1-Cre* mice (Vega et al. 2004). Mice transgenic for Cre in collagen type 2α1-expressing chondrocytes (*Col2α1-Cre*) have been previously reported (Long et al. 2001). These HDAC4^fl/−^, *Col2α1-Cre* mice were subsequently interbred with HDAC4^fl/fl^ animals. Their offspring (*Col2α1-Cre*; HDAC4^d/d^) and their littermates (HDAC4^fl/fl^) were analyzed. Their littermates, HDAC4^fl/fl^ animals, were used as the control. Routine mouse genotyping was performed with the following forward and reverse primers for the Cre allele and the *HDAC4* allele: 5′-ATCCGAAAAGAAAACGTTGA-3′ and 5′-ATCCAGGTTACGGATATAGT-3′, and 5′-ATCTGCCCACCAGAGTATGTG-3′ and 5′-CTTGTTGAGAACAAACTCCTGCAGCT-3′, respectively in each case. The expected product sizes were: 620 bp for the Cre allele, 480 bp for the wild-type *HDAC4* allele, and 480 bp and 620 bp for the floxed *HDAC4* allele.

We divided the experiment into two groups (HDAC4^fl/fl^ control group and *Col2α1-Cre*; HDAC4^d/d^ group). There were 8 mice in each group. Mice were euthanized at postnatal days 14 and 21 for specimen collection.

### Radiography

Mice were anesthetized by 25 mg/kg xylazine and 75 mg/kg ketamine. X-rays were taken with Faxitron MX-20 Cabinet X-ray (Faxitron, Arizona, USA) and used to evaluate skeletal development.

### Cell culture and transfections

Chondrocyte cells were isolated from the ventral parts of rib cages that were resected from 6-day-old mice (C57Bl/6). The cells were subsequently cultured in F-12 media supplemented with 10% fetal bovine serum (FBS; Gibco BRL, USA) at 37 °C in 5% CO_2_ as previously described (Chen et al. 2016a). For transfections, the cells were plated in 6-well plates and grown overnight. When the density of the monolayer reached 70–80% confluency, 1 ml of medium containing serum and antibiotics was added to each well 30–60 min before transfection. Meanwhile, 0.5 μg, 1 μg, 1.5 μg, and 2.0 μg of a vector containing GFP fused to *HDAC4* DNA were each combined with 100 μl of serum-free, high glucose DMEM. The preparations were vortexed gently to mix them. In separate tubes, 3.0 μl of GenJetTM reagent (Ver. II) (SignaGen Laboratories, Ijamsville, MD, USA) was added into 100 ul aliquots of serum-free, high glucose DMEM. The latter preparations were added to the former preparations, with gently pipetting performed to mix them. After a 15 min incubation at room temperature, the GenJetTM-DNA complexes were gently added drop-wise into individual wells and then the plates were swirled to provide homogeneous application of the transfection-DNA complexes onto the cells. The transfected cells were then cultured in a humidified incubator under 5% CO_2_ and hypoxia (2% O_2_) (NuAire Autoflow NU8500 Water Jacket CO_2_ incubator, Plymouth, MN, USA). Forty-eight hours later, the percentage of positively transfected cells (e.g., those expressing GFP) was determined for each sample with a fluorescence microscope (E800; Nikon, Tokyo, Japan). Approximately 300 cells from three independent experiments were scored for each sample.

### Western blot analysis

Forty-eight hours after the chondrocytes were transfected with a vector expressing HDAC4, they were washed with ice-cold PBS and lysed in RIPA buffer (50 mM Tris·HCl (pH 8.0), 150 mM NaCl, 5 mM EDTA, 1% NP-40) at 4 °C. After 30 min, the lysates were cleared by centrifugation for 20 min at 4 °C. Total protein in each sample was quantified with a BCA Protein Assay Reagent Kit (Pierce, Rockford, IL, USA). Western blotting was performed according to standard procedures. Briefly, the proteins were electrophoresed in 10% SDS-PAGE gels and then transferred onto polyvinylidene difluoride (PVDF) membranes. The membranes were incubated with anti-HDAC4 (sc-46,672, Santa Cruz Biotechnology, Santa Cruz, CA, USA), anti-actin (Cell Signaling Technology, Danvers, MA, USA), anti-VEGF (Santa Cruz, CA, USA), and anti-Hif1α (Cell Signaling Technology, Danvers, MA, USA) antibodies, with each at a concentration of 0.2 μg/ml. The membranes were subsequently incubated with peroxidase-conjugated mouse anti-goat (sc-2354, Santa Cruz, CA, USA) and goat anti-mouse (sc-2005, Santa Cruz, CA, USA) secondary antibodies (diluted 1:1000) as appropriate. The relative intensities of HDAC4, VEGF, and Hif1α expression were semi-quantitated by densitometry and normalized to levels of β-actin expression by using Image J software (U.S. National Institutes of Health, Bethesda, MD, USA), as previously described (Li et al. 2014).

### Real-time quantitative PCR (qPCR)

RNA was isolated from the chondrocytes, which were transfected with 2.0 μg GFP-HDAC4 for forty-eight hours, with RNAqueous kit (Ambion, Austin, TX). After treatment with TURBO DNase (Ambion), 1 μg of RNA was reverse-transcribed with random hexamers to obtain first-strand cDNA using iScript cDNA kit (Bio-Rad). The quantification of mRNA for Hif1α and VEGF was performed by two-step real time quantitative RT-PCR (Qiagen, Valencia, CA) (*n* = 3). Simultaneously, controls were conducted in the absence of HDAC4. Primer pairs were as following: for Hif1α, ACA-GTG-GTA-CTC-ACA-GTC-GG (forward) and CCC-TGC-AGT-AGG-TTT-CTG-CT (reverse), for VEGF, ACC-AGC-GCA-GCT-ATT-GCC-GT (forward) and CAC-CGC-CTT-GGC-TTG-TCA-CA (reverse), for 18S, CGG-CTA-CCA-CAT-CCA-AGG-AA (forward) and GCT-GGAATT-ACC-GCG-GCT (reverse). Relative transcript levels were calculated according to the equation x = 2-△△Ct, where △△Ct = △Ct E - △Ct C, △Ct E = Ctexp - Ct18S, and △Ct C=Ct C-Ct 18S) (Fang et al. 2001; Wang et al. 2014a).

### Enzyme-linked Immunosorbent assay (ELISA)

Aliquots of medium from each transfected well were collected and levels of VEGF were measured with a VEGF Immunoassay kit (R&D Systems, Minneapolis, MN, USA) according to the manufacturer’s instructions (Sun et al. 2009).

### Histology

Right knee joints were harvested from animals which were euthanized on postnatal days 14 and 21. The knee joints were fixed in 4% neutral buffered formalin for 24 h at 20 °C and then were processed without decalcification and embedded in a single block. Sections were cut from the sagittal plane, with adjacent sections collected at intervals of 0, 100, and 200 μm from the cartilage layer. Two serial 6-μm-thick sections from each interval were stained with Safranin O and then mounted on slides in order to evaluate development of the growth plate and secondary ossification center. Thickness of the growth plate and ratio of the area of secondary ossification center to the area of tibial plateau were subsequently quantitated from images collected with a Nikon Ri 1 microscope (Nikon, Melville, NY, USA).

### Immunohistochemistry

Briefly, 6-μm sections of knee joint tissue were placed on positively charged glass slides (Thermo Fisher Scientific, Asheville, NC, USA) and then dried with a hotplate to increase the adherence of the tissues to the slides. After the samples were deparaffinized and rehydrated according to conventional methods, endogenous peroxidases were blocked with 3% hydrogen peroxide in methanol (Sigma-Aldrich, Burlington, USA). After 30 min the samples were digested with 5 mg/ml hyaluronidase in phosphate buffered saline (PBS) (Sigma-Aldrich) for 20 min and then were incubated with anti-CD31 (1:100 dilution) (SC-56; Santa Cruz, CA, USA) and anti-CD34 (1:100 dilution) (Santa Cruz, CA, USA) antibodies overnight at 4 °C. Following treatment of the samples with appropriate biotinylated secondary antibodies, a 3,3’diaminobenzidine (DAB) streptavidin-peroxidase (SP) DAB Histostain-SP immunohistochemistry kit (ZYMED203 Laboratories/Invitrogen, Carlsbad, CA, USA) was used to visualize bound antibodies. After the sections were counterstained with hematoxylin (ZYMED Laboratories/Invitrogen), the tissues were imaged with a Nikon Ri 1 microscope (Nikon, Melville, NY, USA) (Guan et al. 2012).

### Statistical analysis

GraphPad 6 software (GraphPad, San Diego, CA, USA) was used to perform statistical analyses.

Data are expressed as the mean ± standard error of the mean (SEM). One-way analysis of variance (ANOVA) Dunnett’s multiple comparisons test was used to compare all data associated with the control and GFP-HDAC4 groups. *P*-values < 0.05 were considered statistically significant.

## Results

### Generation of *Col2α1-Cre*; HDAC4^d/d^ mice

**Fig. 1:**
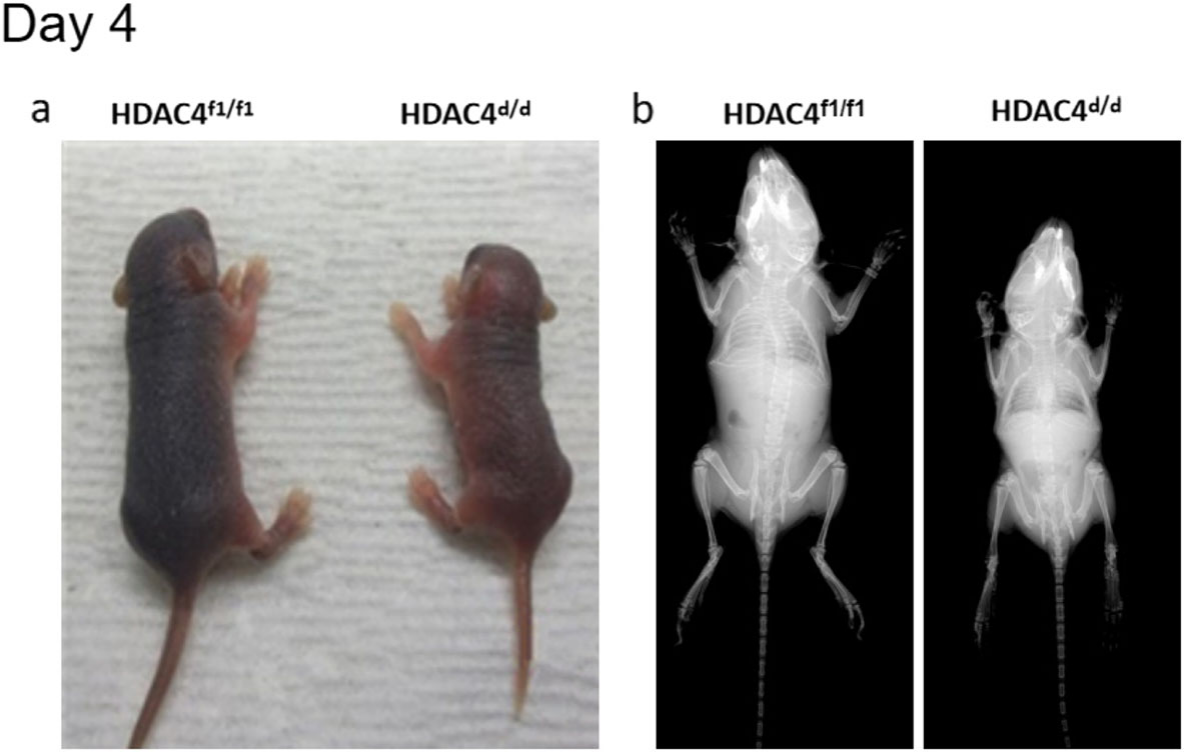
Observations of smaller Col2α1-Cre; HDAC4^d/d^ pups. Photographs (**a**) and X-rays (**b**) of the Col2α1-Cre; HDAC4^d/d^ and HDAC4^f1/f1^ pups taken at postnatal day 4. The Col2α1-Cre; HDAC4^d/d^ mice were markedly smaller

To investigate the function of HDAC4 in vivo, *Col2α1-Cre*; HDAC4^d/d^ mice were used in this study to delete HDAC4 in chondrocytes. Wild-type, homologous, and heterozygous genotypes were confirmed by Southern blot PCR. Growth of the *Col2α1-Cre*; HDAC4^d/d^ mice was subsequently compared with wild-type (HDAC4^f1/f1^) mice at postnatal days 2, 4, 6, and 8, with x-rays taken to examine skeletal development. The *Col2α1-Cre*; HDAC4^d/d^ mice were markedly smaller compared to the HDAC4^f1/f1^ mice (the control group) (Fig. 1), indicating that *Col2α1-Cre*; HDAC4^d/d^ mice undergo slower development when *HDAC4* is disrupted.

### *Col2α1-Cre*; HDAC4^d/d^ mice have extremely shortened growth plates and enlarged secondary ossification centers

**Fig. 2:**
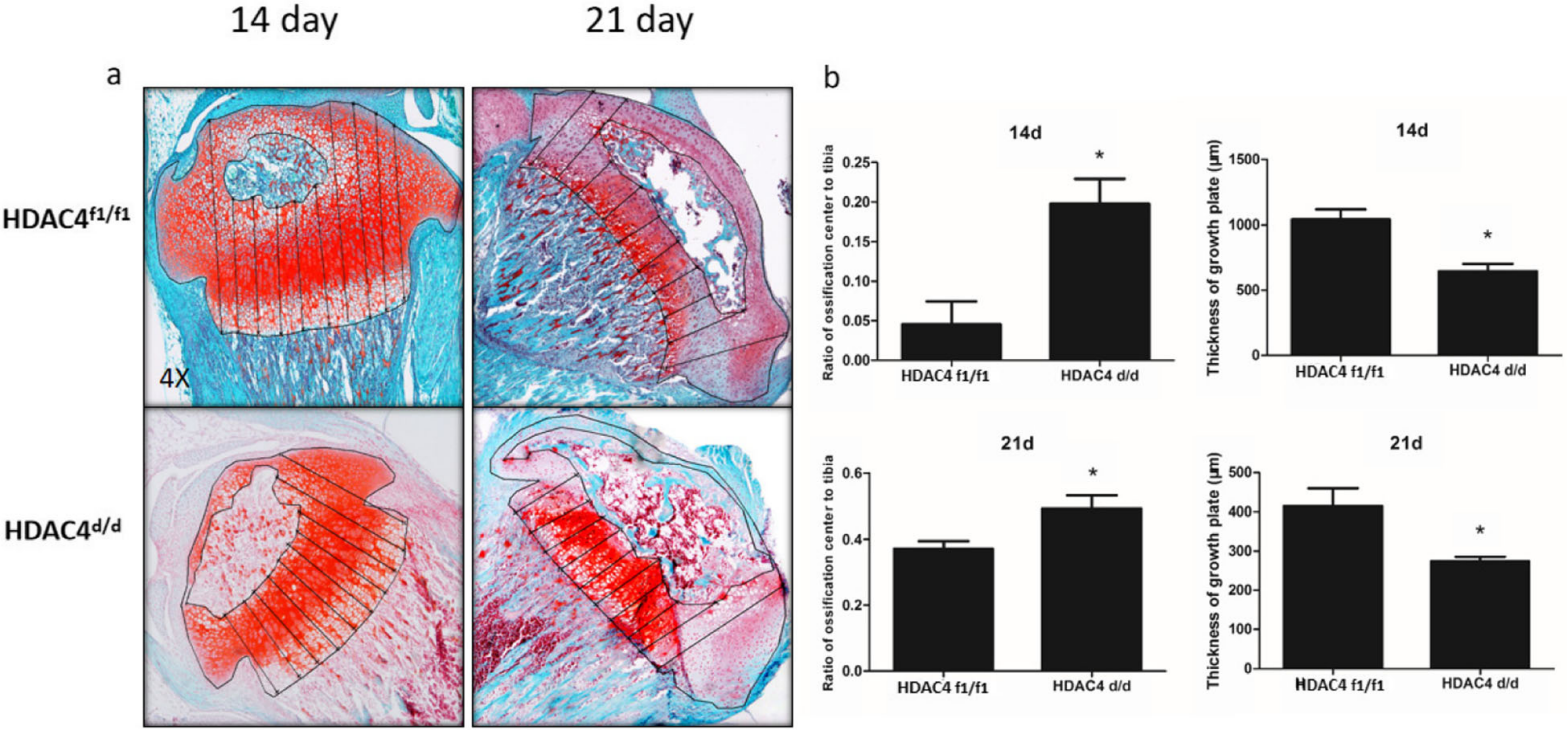
Shortened growth plates and larger secondary ossification centers in Col2α1-Cre; HDAC4^d/d^ mice. Safranin O/Fast green staining was performed on paraffin sections at days 14 and 21. **a** Shortened growth plates and larger secondary ossification centers were observed in the *Col2α1-Cre;* HDAC4^d/d^ mice compared with the HDAC4^f1/f1^ mice on postnatal days 14 and 21. **b** Significant differences in the thicknesses of the growth plates and ratio of area of secondary ossification center to area of tibial plateau were observed between the *Col2α1-Cre*; HDAC4^d/d^ and HDAC4^f1/f1^ groups on postnatal days 14 and 21. Data are expressed as mean ± SEM. *P < 0.05 vs. the vehicle group

Safranin O/Fast green staining was performed on paraffin sections of knee joints resected from *Col2α1-Cre*; HDAC4^d/d^ and HDAC4^f1/f1^ mice at postnatal days 14 and 21. The *Col2α1-Cre*; HDAC4^d/d^ mice exhibited a shortened growth plate and larger secondary ossification center compared with the HDAC4^f1/f1^ mice (Fig. 2a and b). It is noted that the reduction of thickness in three zones (resting, proliferating and hypertrophic) of growth plate was significant for the *Col2α1-Cre*; HDAC4^d/d^ mice at postnatal days 14. HDAC4 knockout has small effect on the thickness reduction of different zones at postnatal days 21.

### Immunohistochemistry of *Col2α1-Cre*; HDAC4^d/d^ mice detected stronger CD31 and CD34 expression

**Fig. 3:**
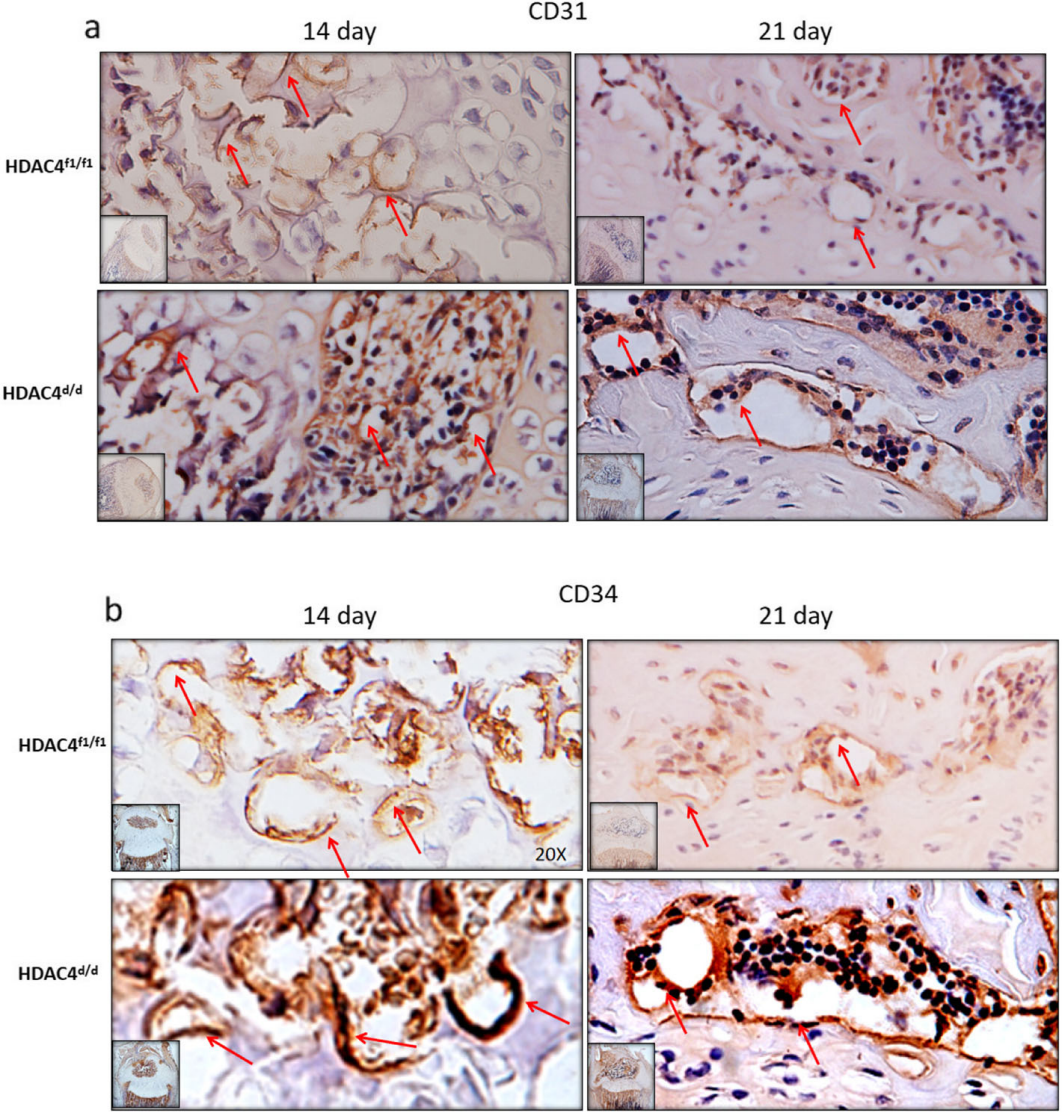
Increased expression of CD31 and CD34 in Col2α1-Cre; HDAC4^d/d^ mice. Strong staining of CD31 (**a**) and CD34 (**b**) was observed in knee joint tissue sections from *Col2α1-Cre*; HDAC4^d/d^ mice compared with knee joint sections from control animals on postnatal days 14 and 21. The red arrow indicates the newly formed vessels

Sections of knee joint tissues collected from *Col2α1-Cre*; HDAC4^d/d^ and HDAC4^f1/f1^ mice on postnatal days 14 and 21 were subjected to immunohistochemistry assays to detect expression of CD31 and CD34. Both proteins exhibited stronger staining in the *Col2α1-Cre*; HDAC4^d/d^ sections than in the control sections (Fig. 3), and the stronger staining appeared in the secondary ossification center of *Col2α1-Cre*; HDAC4^d/d^ mice. These results suggest that vascular invasion is more pronounced when *HDAC4* is disrupted in *Col2α1-Cre;* HDAC4^d/d^ mice.

### Overexpression of HDAC4 in vitro

**Fig. 4:**
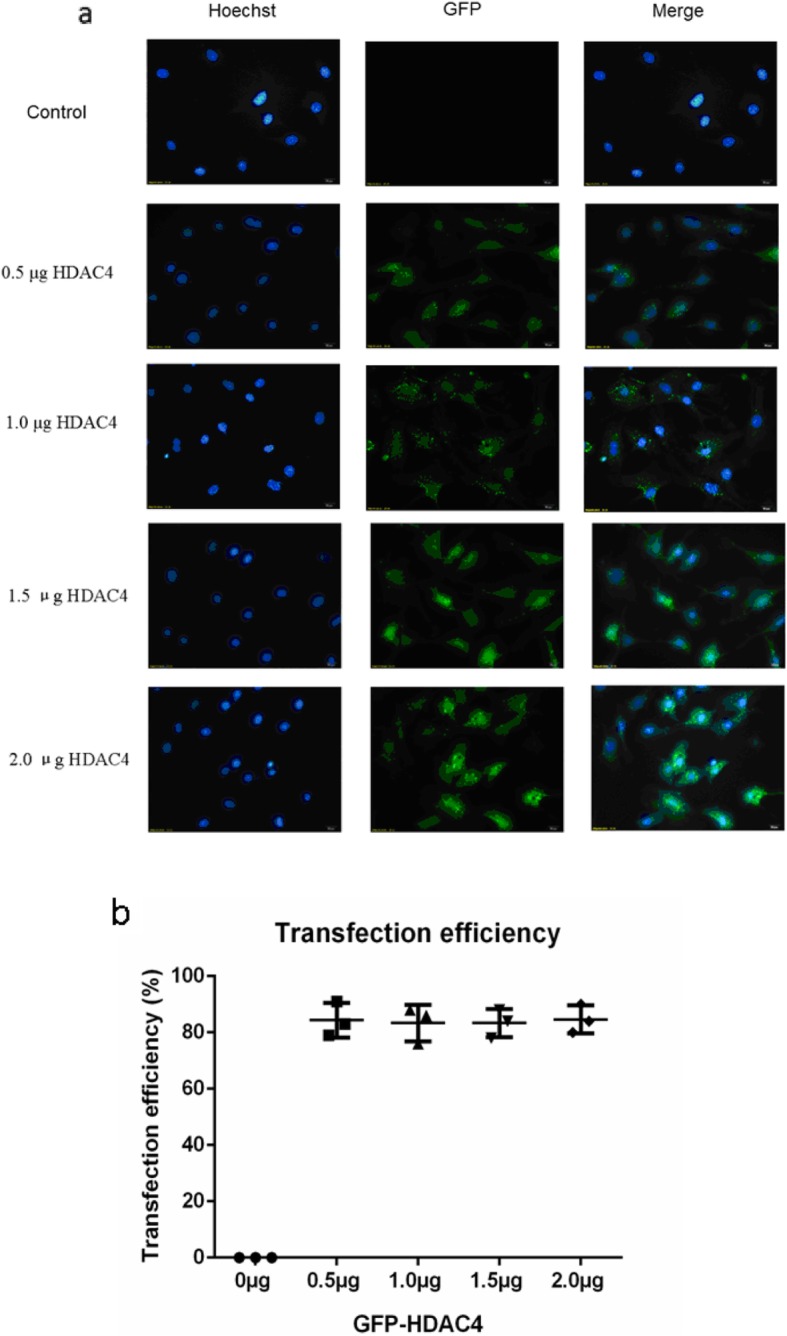
Transfection efficiency of HDAC4 into isolated chondrocytes. **a** The transfection efficiency of various doses of GFP-HDAC4 DNA into isolated murine chondrocytes was examined with confocal laser scanning microscopy based on GFP expression. Nuclei are stained with Hoechst 33342 (Hoechst). **b** Approximately 300 cells from three independent experiments were scored. Data are expressed as mean ± SEM

To establish an in vitro model of HDAC4 overexpression, chondrocyte cells were isolated from the ventral parts of rib cages that were resected from 6-day-old mice (C57Bl/6). After these cells were established as in vitro cultures, the cells were transfected with various doses of a vector expressing HDAC4 as a fusion protein with GFP. Transfection efficiency was evaluated 48 h after transfection based on the numbers of chondrocyte cells expressing GFP. The percentages of GFP expressing cells according to GFP-HDAC4 dose were: 84.3% (79–91%) for 0.5 μg, 83.3% (76–88%) for 1.0 μg, 83.3% (78–88%) for 1.5 μg, and 84.6% (80–90%) for 2.0 μg (Fig. 4).

### Transfection of GFP-HDAC4 leads to a decrease in levels of VEGF and Hif1α

**Fig. 5:**
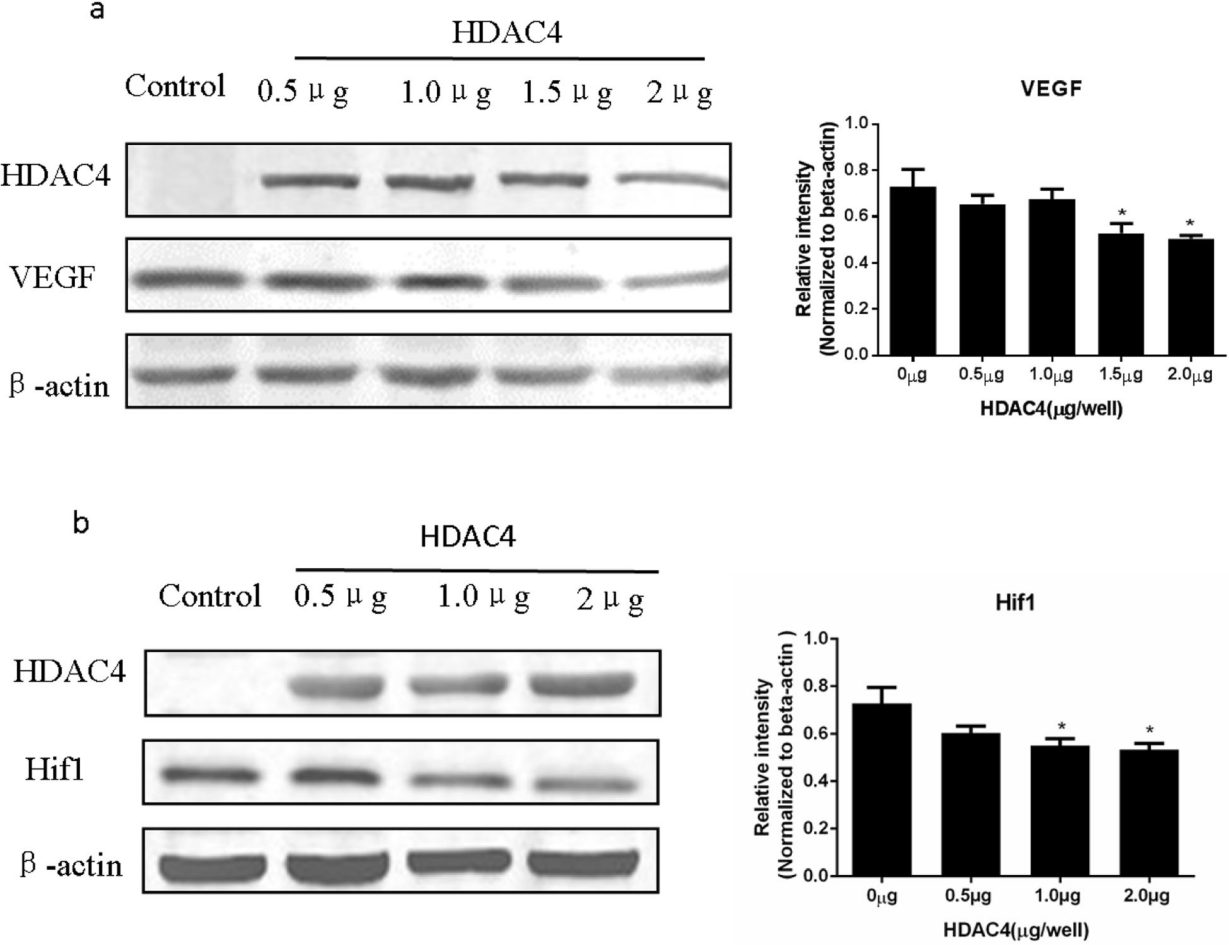
Levels of HDAC4, VEFG, and Hif1α following HDAC4 overexpression. **a, b** Expression levels of HDAC4, Hif1α, and VEGF proteins were detected in Western blots following the transfection of various doses of GFP-HDAC4 DNA into isolated murine chondrocytes. Detection of β-actin was performed as a loading control. The accompanying bar graphs present the average relative intensity values for VEGF and Hif1α, respectively, from three independent experiments. Data are expressed as mean ± SEM. *P < 0.05 vs. vehicle group

Western blotting was performed to detect the levels of HDAC4, VEGF, and Hif1α proteins in chondrocyte cells following overexpression of HDAC4 (Fig. 5a, b). Relative expression levels of VEGF exhibited a significant decrease in the chondrocyte cells that were transfected with 1.5 μg GFP-HDAC4 or 2.0 μg GFP-HDAC4 compared with the control group. Relative expression levels of Hif1α were also significantly lower in the chondrocyte cells transfected with 1.0 μg GFP-HDAC4 and 2.0 μg GFP-HDAC4 compared with the control cells.

**Fig. 6:**
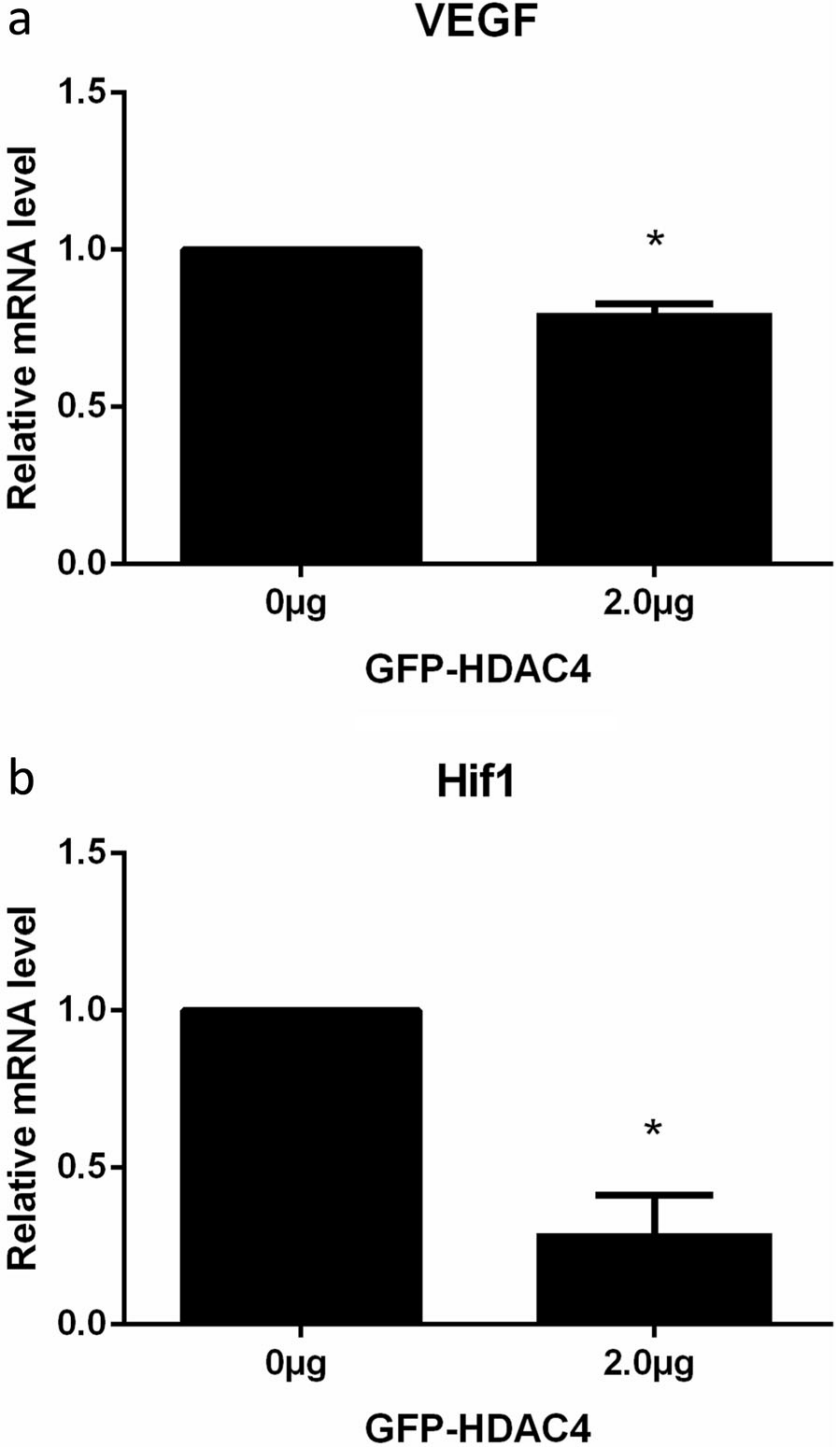
mRNA levels of VEFG and Hif1α following HDAC4 overexpression. **a**, **b** mRNA levels of Hif1α and VEGF were detected in RT-qPCR following the transfection of 2.0 μg GFP-HDAC4 into isolated murine chondrocytes. The accompanying bar graphs present the average relative mRNA values for VEGF and Hif1α, respectively, from three independent experiments. Data are expressed as mean ± SEM. *P < 0.05 vs. vehicle group

The effect of HDAC4 overexpression on the mRNA level of VEGF and Hif1α was determined by RT-qPCR (Fig. 6a, b). The results indicated that the relative mRNA levels of VEGF and Hif1α reduced in the chondrocytes transfected with GFP-HDAC4 compared with the control group.

### Overexpression of HDAC4 leads to a decrease in VEGF level in the media

**Fig. 7:**
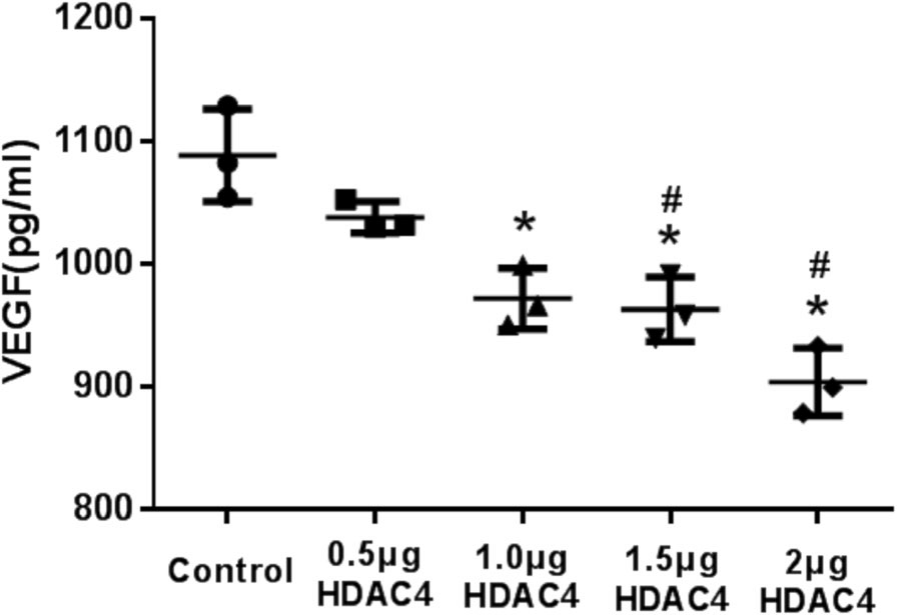
ELISA analysis of VEGF in media. VEGF content in media following the transfection of various doses of GFP-HDAC4 DNA into isolated murine chondrocytes as detected by ELISA. Data are expressed as mean ± SEM. *P < 0.05 vs. vehicle group. #*P* < 0.05 vs. 0.5 μg HDAC4 group

Forty-eight hours after transfection with GFP-HDAC4, samples of media were collected to detect levels of VEGF. The ELISA data indicated that increased expression of HDAC4 led to a decrease in the amount of VEGF into the media (Fig. 7). Moreover, the difference in the amount of secreted VEGF compared with the control group was significant for the 1.0 μg GFP-HDAC4, 1.5 μg GFP-HDAC4, and 2.0 μg GFP-HDAC4 groups.

## Discussion

During skeletal development, the appendicular skeleton and part of the axial skeleton are formed largely by endochondral ossification. The latter is a sequential process that involves the proliferation, differentiation, cell death, and resorption of chondrocytes. Skeletal vasculature plays a significant role in the process of bone development (including endochondral and intramembranous ossification), as well as in the regeneration and remodeling of bone (Dai and Rabie 2007; Hankenson et al. 2011; Huang et al. 2016a). Blood vessels supply bones with needed nutrients, including oxygen, hormones, and growth factors, thereby regulating the process of bone development and remodeling (Filipowska et al. 2017). Secretion of VEGF by chondrocytes is also essential for the coupling of osteogenesis and angiogenesis (Hu and Olsen 2016). HDAC4 has been shown to be a critical negative regulator of chondrocyte hypertrophy based on its ability to bind and inhibit Runx2, a transcription factor that is critical for chondrocyte hypertrophy (Zhou et al. 2015) and whose expression promotes both bone metastasis and osteolysis (Trotter et al. 2015). To date, there is no direct evidence that expression of HDAC4 decreases blood vessel formation in the growth plate, although it has been demonstrated that global deletion of HDAC4 is lethal in mice (Vega et al. 2004). To examine the significance of chondrocyte-derived HDAC4 in angiogenesis, we selectively ablated the *HDAC4* gene from collagen type II-expressing cells by using Cre/loxP gene targeting. As a result, severely runted and slow-growing mice were generated. Moreover, compared with the HDAC4^f1/f1^ group, shortened growth plates were observed with Safranin O/Fast green staining and a larger ratio of ossification center to tibia was measured for the *Col2α1-Cre;* HDAC4^d/d^ mice by postnatal days 14 and 21. The similar result was also found from Col2-Cre; HDAC4^f1/f1^ mice at birth by Nishimori et al., and however the Col2-Cre; HDAC4^f1/f1^ mice died around days 14 (Nishimori et al. 2019). Increased expression of CD31 and CD34 was detected in the knee joints of the *Col2α1-Cre;* HDAC4^d/d^ mice by postnatal days 14 and 21 as shown in Fig. 3a and b. Both CD31 and CD34 are widely used as markers of angiogenesis, hematopoietic cells, and endothelial progenitor cells (Hassanein et al. 2017). Thus, the present data are consistent with a role for *HDAC4* in both angiogenesis and osteoblast differentiation via regulation of Runx2 and osteocalcin expression (Sun and Beier 2014; Lawson et al. 2013).

VEGF is an important mediator of angiogenesis (Dai and Rabie 2007), while Hif1α is a transcription factor that stimulates the growth of new blood vessels during angiogenesis (Filipowska et al. 2017). It has been shown that vascular endothelial cells that are localized near the chondro-osseous junction express higher levels of Hif1α and exhibit strong expression of CD31 (Kusumbe et al. 2014). When HDAC4 was overexpressed in isolated murine chondrocytes, a significant decrease in VEGF and Hif1α protein expression was detected by Western blot. The HDAC4 overexpression also repressed the mRNA level of VEGF and Hif1α as determined by RT-qPCR. In addition, a significant decrease in the secretion of VEGF was further detected by ELISAs. However, this was inconsistent with the previous reports, in which Ablating HDAC4 signaling induced Hif1α protein acetylation, reduced Hif1α protein level in cancer cell lines (Geng et al. 2011; Isaacs et al. 2013; Ellis et al. 2009), and inhibited endothelial tumor angiogenesis (Geng et al. 2011; Cadot et al. 2009; Liu et al. 2009; Mottet et al. 2009). One of the reasons may be related to the cell type. In the previous reports, the relationship of HDAC4 and Hif1α was investigated in cancer cell lines or for tumor angiogenesis. In the present study, the chondrocytes that were transfected with higher doses of HDAC4 vector exhibited lower levels of VEGF and Hif1α expression compared with the cells that were transfected with lower doses of HDAC4 vector. The present results are consistent with our CD31 and CD34 straining results in which specific deletion of HDAC4 from chondrocytes in vivo led to a strong staining of CD31 and CD34. These results verify that HDAC4 is a critical regulator of angiogenesis and development of cartilage growth plates, and they support our hypothesis that overexpression of HDAC4 may inhibit angiogenesis in growth plates by decreasing levels of VEGF and Hif1α. However, it is noted that VEGF/ Hif1α is not the only factor promoting angiogenesis. There may be other unknown ways involving the results due to deletion of HDAC4. Gene array is going to be conducted to explore the unknown pathway in the future study.

## Conclusions

In the present study, selective gene targeting was achieved to specifically ablate the *HDAC4* gene from collagen type 2α1-expressing cells. Following this ablation, the affected mice exhibited a remarkable phenotype which included a shorter growth rate, enhanced angiogenesis, the formation of premature bone, and an enlarged secondary ossification center. Correspondingly, our in vitro results also demonstrated a role for HDAC4 in angiogenesis, and they identified VEGF and Hif1α as downstream targets.

## Data Availability

All relevant data supporting the conclusions of this article is included within the manuscript.

## Abbreviations

BMP2: Bone morphogenetic protein 2
ELISA: Enzyme-linked immunosorbent assay
FGF-2: Fibroblast growth factor 2
GFP: Green fluorescent protein
HDAC4: Histone deacetylase 4
Hif1α: Hypoxia-inducible factor 1α
IHH: Indian hedgehog
MMP-13: Matrix metalloproteinase 13
Runx2: Runt-related transcription factor-2
VEGF: Vascular endothelial growth factor
